# Reduced spermatozoa functionality during stress is the consequence of adrenergic-mediated disturbance of mitochondrial dynamics markers

**DOI:** 10.1038/s41598-020-73630-y

**Published:** 2020-10-08

**Authors:** Isidora M. Starovlah, Sava M. Radovic Pletikosic, Tatjana S. Kostic, Silvana A. Andric

**Affiliations:** Laboratory for Reproductive Endocrinology and Signaling, Laboratory for Chronobiology and Aging, Center of Excellence for Reproductive Endocrinology (CeRES), DBE, Faculty of Sciences, University of Novi Sad, Dositeja Obradovica Square 2, 21000 Novi Sad, Serbia

**Keywords:** Reproductive biology, Cell signalling

## Abstract

Here we investigate the stress-signaling responsible for the effects of acute/repeated psychological stresses (the most common stresses in human society) on spermatozoa number and functionality, as well as the transcriptional profile of mitochondrial dynamics markers by using the in vivo and ex vivo approaches. Acute and repeated stress inhibit spermatozoa functionality (acute –> 3.2-fold, repeated –> 2.5-fold), while only repeated stress reduces the spermatozoa number (1.7-fold). Stress hormones mimic these effects and decrease the spermatozoa functionality (adrenaline: 10 µM –> 2.4-fold, 100 µM – > 2.8-fold; hydrocortisone: 50 pM –> 2.7-fold, 500 pM –> 8.5-fold). They also significantly disturb the transcriptional profile of all main mitochondrial dynamics markers in spermatozoa. Ex vivo manipulation of stress signaling in spermatozoa reveals that most of these effects are mediated through ɑ1-and/or-β-adrenergic receptors. The transcription of these receptors and their kinases in the same samples is under the significant influence of adrenergic signaling. Our results are the first to show the importance of mitochondrial dynamics markers in spermatozoa since the transcriptional profiles of sixteen-out-of-ninteen are disturbed by manipulation of stress-hormones-signaling. This is a completely new molecular approach to assess spermatozoa functionality and it is important for a better understanding of the correlations between stress, environmental-life-style and other factors, and male (in)fertility.

## Introduction

A growing body of evidence, states the increasing rate of male infertility in humans, an increasing number of unexplained cases of infertile males in the peak of the reproductive period, and a decrease of the fertility rate in men younger than age 30. The many studies discussed the correlation between male (in)fertility and stress and/or stressful life^1,2^. Besides, the semen quality and male fertility are important not only as of the fundamental marker of reproductive health but also as the fundamental biomarkers of overall health and harbingers for the development of comorbidity and mortality. However, the exact nature of these associations remains somewhat unclear, although hypothesized mechanisms include genetic, developmental, and lifestyle-based factors^3^. It has been suggested that immobilization stress can enhance testicular germ cell apoptosis in rats^4^. Besides, stress-induced loss of germ cells in rats leads to a decrease in sperm count due to oxidative damage caused by chronic stress and the majority of these changes are not reversible^5^. It is well recognized that stress is a major contributor to the wide variety of psychosocial and physical pathological conditions in humans^6–10^. Different types of stressors, including occupational stress, examination stress and, major stressful life events (including fertility treatment) have been linked to reduced adult male reproductive function. The previous studies showed a decline in semen quality during fertility treatment and the infertile men reported a higher number of stressful life events than fertile men^2^.

During stress, an orchestrated adaptive compensatory specific response of the organism (so-called “fight and flight” response) is activated to sustain homeostasis^11^. The main hallmarks of stress are increased levels of circulating stress hormones/mediators, including glucocorticoids (GCs) and the catecholamines^6,10–13^ as well as decrease of circulating testosterone in males^14–17^. At the cellular level, stressors can strongly disturb cellular homeostasis and some of the effects could be inherited. It has been shown that sperm RNA carries marks of trauma^18^ and that it is involved in the transgenerational inheritance of the effects of early trauma in mice^19^. Epidemiological studies strongly suggested that stress-induced DNA damage may promote various diseases and that a stress-response-β2-adrenergic-receptors(β2-ADRs)-pathway regulates DNA damage^20^. ADRs can activate the biogenesis of new mitochondria, the key components of the stress response^6,8,9^, but also for sperm functionality^21^. Mitochondria are primarily responsible for meeting the enormous energy demands of the ”fight and flight response” by using the large amounts of substrates that are made available by stress hormone-induced mobilization from energy storages^6,8,9^.

For spermatozoa functionality, both, ADRs and mitochondria are essential. Fertility and spermatogenesis are altered in α1-ADRs-knockout-male-mice^22^. The functionality of human sperm mitochondria differentiates human spermatozoa with high and low fertilizing capability^23^. It has been suggested that mtDNA depletion may play an important role in the pathophysiology of male infertility^24^ and serve as useful diagnostic markers of sperm quality in infertile men^25^. The mitochondrial morphological changes are specific for stages of spermatogenesis^26^ and could explain the strong association of altered ultrastructure of mitochondria with unexplained asthenozoospermia^27^. Clinical trials showed that the mtDNA of oligo-asthenozoospermic patients present some defects that made DNA unavailable for amplification^28^ and that large-scale deletions of mtDNA may be genetic risk factors for poor sperm quality in asthenoteratozoospermia-induced male infertility^29^. Numerous studies on humans pointed the importance of mitochondrial membrane potential not only for spermatozoa functionality^30–32^ but also, in combination with sperm DNA fragmentation, as a superior to standard semen parameters for the prediction of natural conception^33^. It has been shown that TFAM is associated with the reduction in mtDNA content of human sperm^34^ and that TFAM gene expression positive correlate with abnormal forms, sperm DNA fragmentation and mtDNA copy number^35,36^. Besides, mitophagy may be regulating human sperm function such as motility and viability^37^, UCP2 mitigates the loss of human spermatozoa motility^38^, while the expression level of MFN2 is related to motility and cryoprotective potentials of human sperm^39^. Accordingly, the mitochondria are a key organelle for sperm motility and strongly correlate with (in)fertility^21^. However, there is no, according to the best of our knowledge, any published pieces of evidence about the profile of mitochondrial dynamic markers, although profiling of signaling proteins in human spermatozoa indicated that the phosphorylated levels of several proteins were significantly correlated with motility parameters^40^ and these proteins are involved in the regulation of mitochondrial-network-homeostasis.

The homeostasis of mitochondrial network is maintained by intriguing, but well-coordinated processes of mitochondrial dynamics. To preserve and protect their functional status, mitochondria can maintain the complex mitochondrial protein-import machinery (mitochondrial transduceom), position themselves strategically in the cell (motility/trafficking), unite (fusion), divide (fission), make the pool of new and healthy mitochondria (mitochondrial biogenesis) and if irreversibly damaged or dysfunctional eliminated (mitophagy)^41–44^. All mentioned processes are a complex sophisticated and multistep interplay of cellular and molecular events that cells use to renews, adapts, or expands its mitochondrial population arranged in the network during episodes of damage or periods of intensified energy demand^44–47^. The spatiotemporal regulation of mitochondrial dynamics is achieved by the nucleo-mitochondrial interactions dependent on the interplay between transcription factors and members of the PGC1 family of coactivators (PGC1α, PGC1β, PRC) regulating the expression of the main markers of mitochondrial dynamic^43,44^. This includes the main markers of mitochondrial fusion (MFN1, MFN2, OPA1), fission (DRP1, FIS1), biogenesis (PGC1α, PGC1β, NRF1, NRF2, TFAM) and mitophagy (PINK, PARKIN), but also important markers of the respiratory chain function, the mitochondrial transcription-translation-replication machinery, and protein import-assembly apparatus^41–45,47^. These dynamic processes are controlled by an array of mitochondrial and cellular signaling pathways^44–46^ which convey environmental signals including temperature^48^, energy deprivation^45^, stress^49,50^, availability of nutrients^45^ and growth factors^51^. It is very important to point out that all signaling pathways regulating mitochondrial dynamics are deeply involved in the regulation of spermatozoa function.

Here we hypothesize that the stress alters the signaling pathways and molecules responsible for processes of mitochondrial dynamics and architecture in spermatozoa with consequences for the function. Besides, these markers may serve as a new diagnostic tool. The immobilization stress (IMO) was chosen as a typical and frequently used model of psychophysical stress^10,12,13,17^. The focus of the present study was on the effect of stress-hormones-signaling on the transcriptional profile of mitochondrial biogenesis and fusion/architecture markers and on potential signaling pathways responsible for the regulation of these processes in spermatozoa.

## Results

In the search for the possible mechanism(s) causing the reduced spermatozoa functionality during/after psychological stress, two approaches (in vivo and ex vivo) were applied. The in vivo approach was design to mimic the situations in the human population exposed to acute as well as repeated psychological stress, the most common stress in human society, by using the immobilization of the adult male rat^16,17^. The ex vivo approach was performed on epididymal spermatozoa isolated from the undisturbed adult male rats and exposed to stress hormones and the agonists/antagonists of their receptors. The localization of the main markers of mitochondrial dynamics as well as signaling molecules regulating the mitochondrial network dynamic is included in Supp. Results within the Supp. Information (please see Supp. Figure 1).

**Figure 1:**
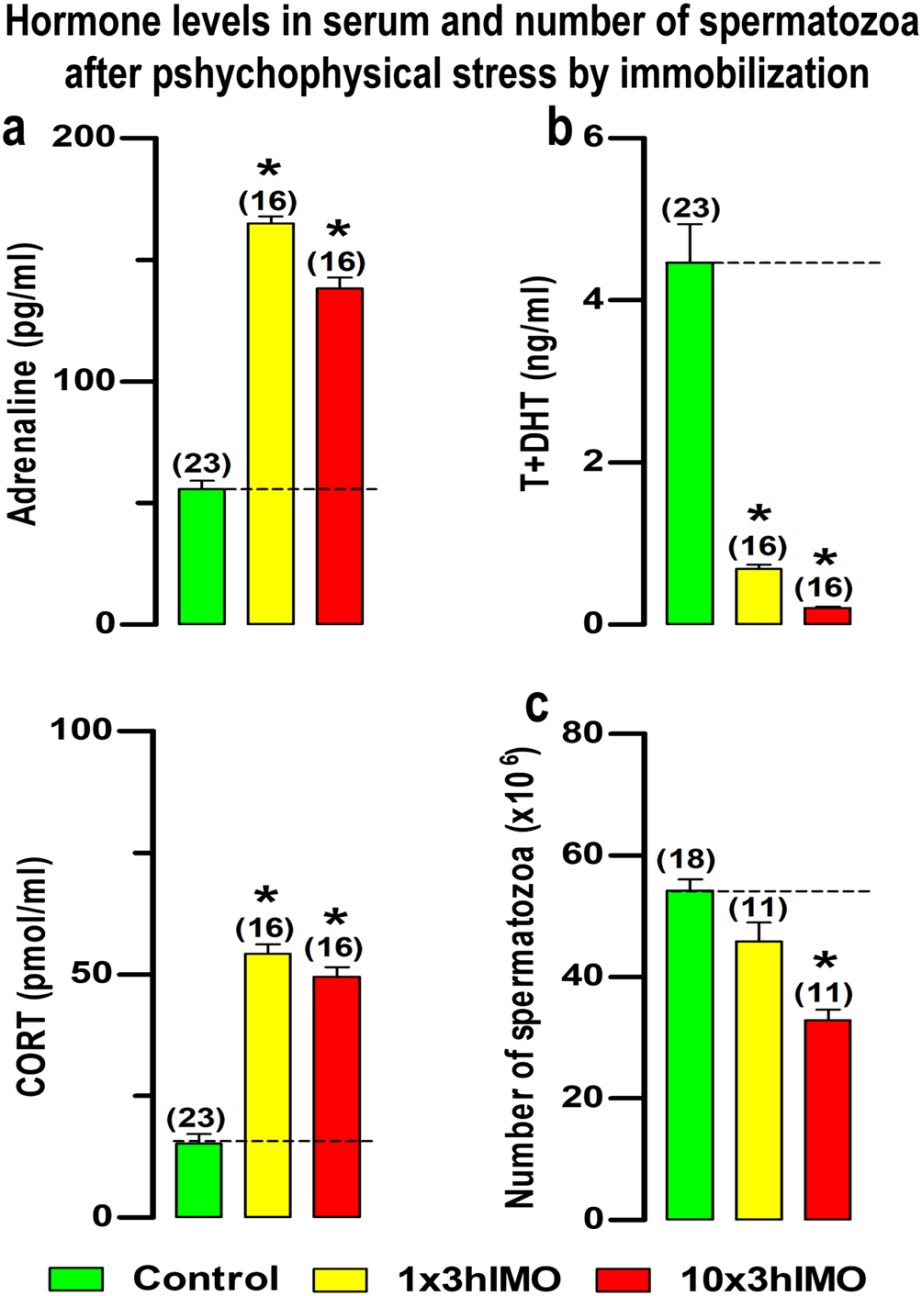
The psychophysical stress by immobilization increases the level of stress hormones in circulation, but decreases androgens levels and the number of spermatozoa. The circulating (serum) levels of (**a**) stress hormones **a**drenaline and corticosterone (CORT), as well as, (**b**) androgens (testosterone + dihydrotestosterone, T + DHT) after psychophysical stress by immobilization. (**c**) The number of spermatozoa isolated from caudal epididymides of unstressed rats (control), rats subjected to acute immobilization (IMO) stress once, for 3 h (1 × 3hIMO) and rats subjected to repeat IMO of 3 h for 10 consecutive days (10 × 3hIMO). Data bars are mean ± SEM values of four independent in vivo experiments and individual isolation of spermatozoa from each rat (please see the number of rats in the brackets). Statistical significance was set at level *p* < 0.05: * vs. control group.

### The psychophysical stress by immobilization increases the level of stress hormones in circulation, but decreases androgens levels and the number of spermatozoa

The effects of the acute (1 × 3hIMO) as well as repeated (10 × 3hIMO) stress were confirmed by measurement of the concentrations of stress hormones (corticosterone and adrenaline), as well as androgens (T + DHT), in serum obtained from undisturbed controls and stressed animals. In agreement with previous studies^16,17^ IMO was effective as a stressor elevating the serum adrenaline (1 × 3hIMO—threefold, 196.8%; 10 × 3hIMO—2.5-fold, 148,4%) and corticosterone (1 × 3hIMO—3.5-fold, 256.1%; 10 × 3hIMO—3.3-fold, 224.7%) levels in all stressed groups, while circulating androgens (T + DHT) were reduced in all stressed rats (1 × 3hIMO—6.5-fold, 84.5%, 10 × 3hIMO—21.3-fold, 95.3%). Besides, only repeated immobilization significantly reduced (10 × 3hIMO—1.7-fold, 39.4%) the number of spermatozoa (Fig. 1c).

### The functionality of spermatozoa decreases after in vivo psychophysical stress and ex vivo stimulation of spermatozoa with stress hormones adrenaline and hydrocortisone

**Figure 2:**
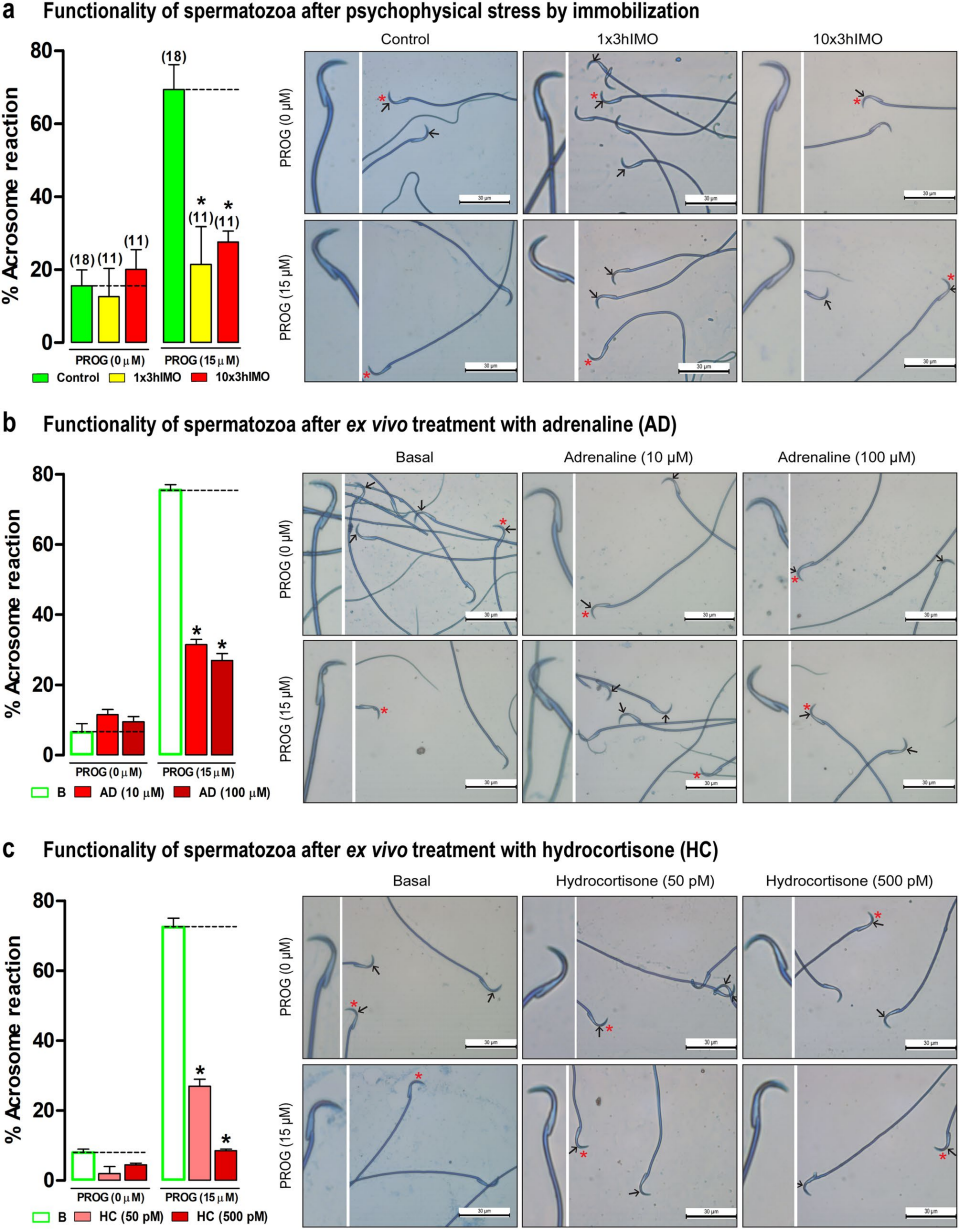
The functionality of spermatozoa decreases after in vivo psychophysical stress and ex vivo stimulation of spermatozoa with stress hormones adrenaline and hydrocortisone. The functionality of spermatozoa (% acrosome reacted spermatozoa) after psychophysical stress by immobilization (**a**), or ex vivo treatment for 30 min with adrenaline—AD (**b**) or hydrocortisone—HC (**c**). Capacitated spermatozoa were stimulated with progesterone (PROG 15 µM) in parallel with spermatozoa not treated with progesterone (PROG 0 µM). Blue staining in the acrosome region of the head indicated intact acrosome, whereas spermatozoa without blue staining in the acrosome region were considered to be acrosome reacted. Arrows indicate acrosome intact spermatozoa; scale bar 30 µm. Star indicate spermatozoa magnified on the right panel. Data are presented as the percentage of acrosome reacted spermatozoa ± SEM values of four independent in vivo experiments and individual isolation and capacitation/acrosome-reaction-processing of spermatozoa from each rat (please see the number of rats in the brackets), while for ex vivo experiments SEM values of three independent experiments involving six rats per experiment (eighteen in total). Statistical significance was set at level *p* < 0.05: * vs. control group (**a**—in vivo); or basal group (**b**, **c**—ex vivo).

Both types of stress, acute and repeated, inhibited spermatozoa functionality (1 × 3hIMO—3.2-fold, 10 × 3hIMO—2.5-fold). The ex vivo application of stress hormones adrenaline and hydrocortisone mimics the effect of stress on spermatozoa functionality (Fig. 2). The stress-hormones-agonists mimic the effect of stress by decreasing the spermatozoa functionality (adrenaline: 10 μM—2.4-fold, 100 μM—2.8-fold; hydrocortisone: 50 pM—2.7-fold, 500 pM—8.5-fold). To reveal the possible mechanism(s) beyond these effects, never explored the markers of mitochondrial dynamics were followed.

### The stress hormones change the transcriptional profile of mitochondrial biogenesis markers in spermatozoa

**Figure 3:**
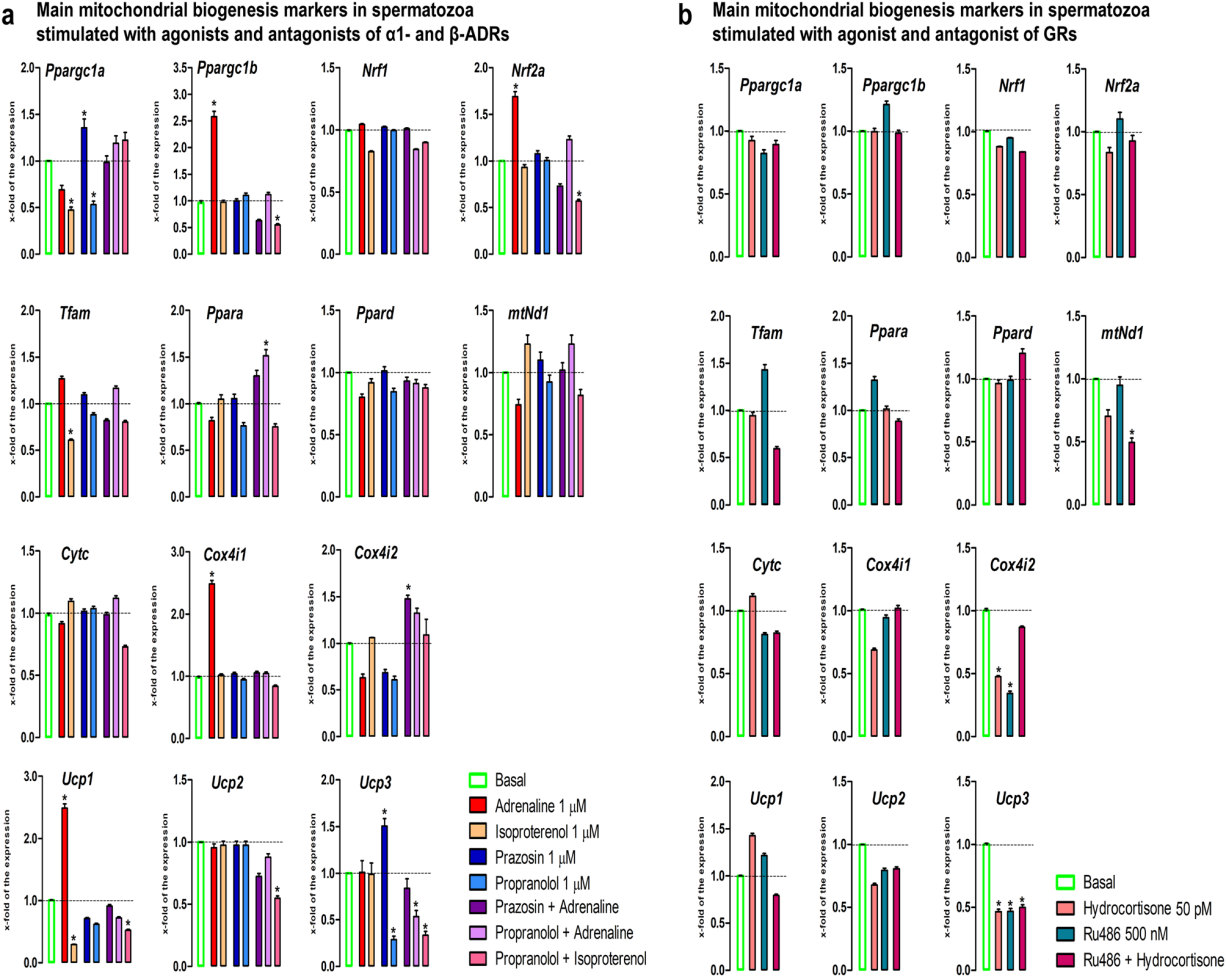
The stress hormones change the transcriptional profile of markers of mitochondrial biogenesis in spermatozoa. Spermatozoa isolated from undisturbed rats were treated ex vivo, for 6 h, either with adrenergic receptors (ADRs) agonists (adrenaline, isoproterenol) or/and antagonists (ɑ1-ADRs antagonists prazosin; β-ADRs antagonists propranolol) or glucocorticoid receptors (GRs) agonist (hydrocortisone) or/and antagonist (RU486). The RNA was used for analyses of the transcriptional profile of mitochondrial biogenesis markers (**a**, **b**). Data bars are mean ± SEM values of three independent ex vivo experiments involving six rats per experiment (eighteen in total). Statistical significance was set at level *p* < 0.05: * vs. basal group.

The stress hormones disturbed the transcriptional profile of the markers of mitochondrial biogenesis (11-out-of-14) (Fig. 3).

The transcription of PGC1, the master regulator involved in transcriptional control of all the processes related to mitochondrial homeostasis and integrator of environmental signals^43,44^, was changed. The level of *Ppargc1a* significantly decreased (2.1-fold) in spermatozoa incubated with isoproterenol, β-ADRs-agonist, but also in those incubated with propranolol (1.9-fold), β-ADRs-antagonist, and both effects were abolished by the combination (propranolol + isoproterenol), suggesting the involvement of β-ADRs. Oppositely, the transcription of *Ppargc1b* in spermatozoa significantly increased (2.6-fold) by adrenaline and this effect was completely abolished with both ɑ1-ADRs-antagonist and β-ADRs-antagonist, suggesting the involvement of both types of ADRs. Besides, the level of *Ppargc1b* transcript decreased by the combination of β-ADRs-antagonist/agonists (propranolol + isoproterenol; 1.8-fold). In the same spermatozoa-samples, the transcriptional profiles of *Nrf1* and *Nrf2a*, PGC1-downstream-targets that act on the genes for OXPHOS subunits^43,44^ were differently regulated. The level of *Nrf1* transcript remained unchanged independently of the type of agonist/antagonist, while *Nrf2a* transcript increased (1.7-fold) by adrenaline, but decreased (1.8-fold) by propranolol + isoproterenol. The effect of adrenaline on *Nrf2a* was completely abolished with both ɑ1-ADRs-antagonist and β-ADRs-antagonist. The level of transcript for *Tfam*, a downstream target of both NRF1 and NRF2, decreased (1.6-fold) by β-ADRs-agonist-isoproterenol, and this effect was abolished by β-ADRs-antagonist-propranolol, suggesting sole involvement of β-ADRs in the regulation of spermatozoa *Tfam*. The *Ppara* transcript-level in spermatozoa significantly increased (1.5-fold) by propranolol + adrenaline-treatment, while the transcriptional profile of *Ppard* in spermatozoa remained unchanged independently of the manipulation of stress-hormone-receptors. The transcriptional profiles of all the above-mentioned markers remained unchanged after treatment with either GRs-agonist-hydrocortisone or GR-antagonist-RU486. However, the level of transcript for *mtNd1*, an mtDNA encoded transcript whose core subunit belongs to the minimal assembly required for catalysis, significantly decreased (twofold) in spermatozoa samples treated with RU486 + hydrocortisone. The transcriptional profiles of other downstream NRF1/NRF2 targets (CytC, COX4) were differently regulated. The transcription of *Cytc* remained unchanged independently of the type of ADRS-agonists/antagonists. The level of *Cox4i1* transcript in spermatozoa rise (2.5-fold) by adrenaline-stimulation and this effect was completely abolished with both ɑ1-ADRs-antagonist and β-ADRs-antagonist. The *Cox4i2* transcript increased (1.5-fold) in spermatozoa incubated with combination prazosin + adrenaline, but decreased in spermatozoa incubated with hydrocortisone (2.1-fold) and RU486 (2.9-fold). Also, stress-signaling significantly changed the transcriptional profile of the genes (*Ucp1,* *Ucp2,* *Ucp3*) for proteins the mediators of regulated proton leak and controllers of the production of superoxide and other downstream reactive oxygen species^48^. The level of *Ucp1* transcript significantly increased (2.5-fold) in spermatozoa incubated with adrenaline and this effect was abolished in the presence of ɑ1-ADRs-antagonist and β-ADRs-antagonist. Oppositely, incubation of spermatozoa with β-ADRs-agonist-isoproterenol or combination-propranolol + isoproterenol caused a decrease (3.4-fold and 1.9-fold respectively). Same combination also decreased (1.8-fold) the level of *Ucp2*. The level of *Ucp3* transcript was significantly reduced in spermatozoa incubated with propranolol (3.5-fold) or propranolol + adrenaline (1.9-fold) or propranolol + isoproterenol (threefold) or hydrocortisone (2.2-fold), or RU486 (2.1-fold) or combination-RU486 + hydrocortisone (twofold).

### The stress hormones change the transcriptional profile of mitochondrial fusion and architecture markers in spermatozoa

**Figure 4:**
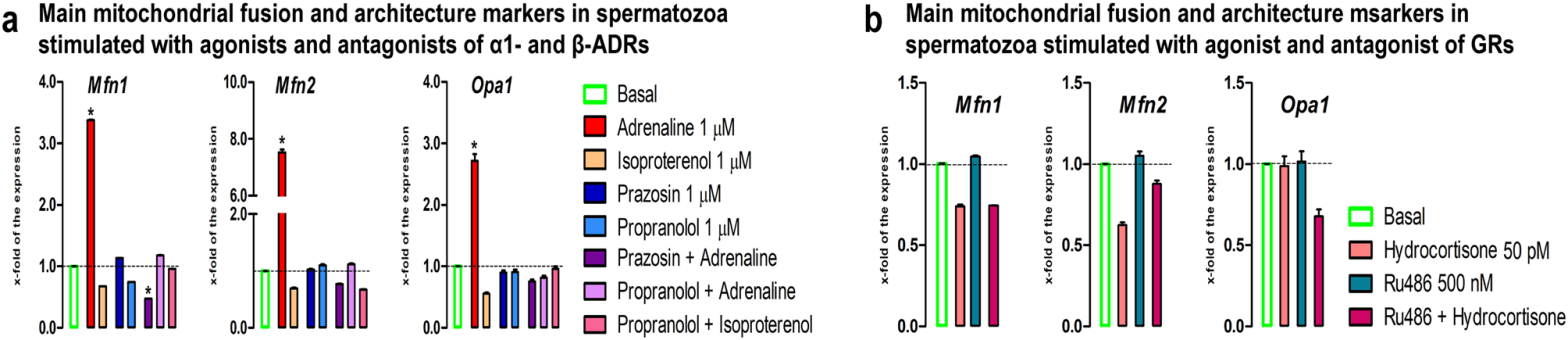
The stress hormones change the transcriptional profile of mitochondrial fusion and architecture markers in spermatozoa. Spermatozoa isolated from undisturbed rats were treated ex vivo, for 6 h, either with adrenergic receptors (ADRs) agonists (adrenaline, isoproterenol) or/and antagonists (ɑ1-ADRs antagonists prazosin; β-ADRs antagonists propranolol) or glucocorticoid receptors (GRs) agonist (hydrocortisone) or/and antagonist (RU486). The RNA was used for analyses of the transcriptional profile of mitochondrial fusion and architecture markers (**a**, **b**). Data bars are mean ± SEM values of three independent ex vivo experiments involving six rats per experiment (eighteen in total). Statistical significance was set at level *p* < 0.05: * vs. basal group.

Giving the central importance of mitochondrial architecture and fusion for homeostasis of mitochondrial function and network^43,44,47^, the main markers (*Mfn1,* *Mfn2,* *Opa1*) of mitochondrial fusion/architectures were followed in spermatozoa. Results showed that the transcriptional profiles of all markers (Fig. 4a,b) were significantly increased by adrenaline (*Mfn1*—3.4-fold; *Mfn2*—7.5-fold; *Opa1*—2.7-fold) and these effects were completely abolished with both ɑ1-ADRs-antagonist and β-ADRs-antagonist, suggesting the involvement of both types of ADRs. Also, the level of *Mfn1* transcript significantly decreased (2.1-fold) in spermatozoa incubated with combination-prazosin + adrenaline, suggesting that ɑ1-ADRs could mediate more stimulatory effects.

### The stress hormones change the transcriptional profile of mitochondrial fission markers in spermatozoa

**Figure 5:**
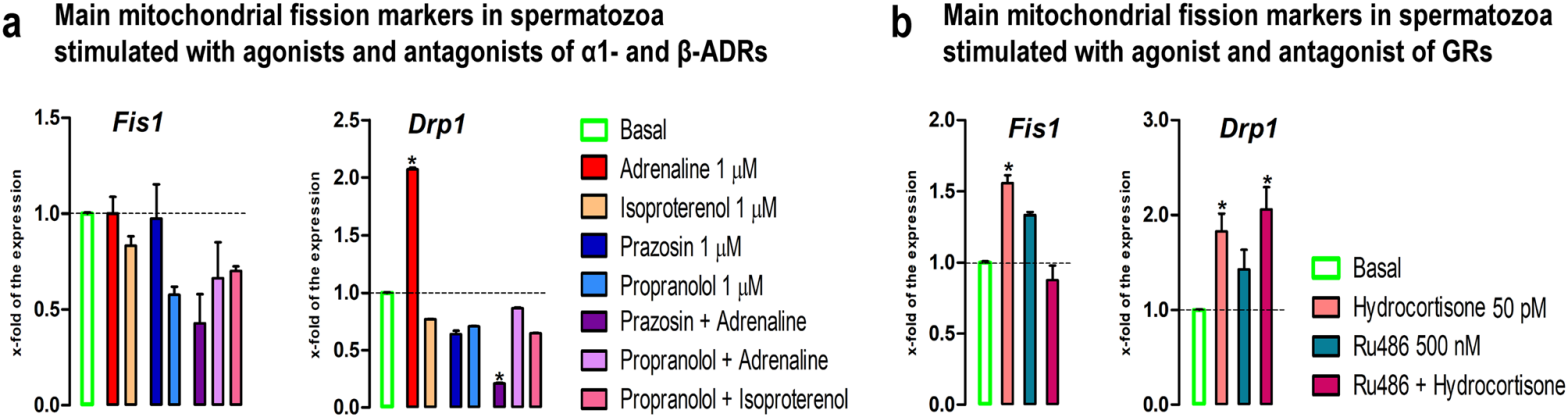
The stress hormones change the transcriptional profile of mitochondrial fission markers in spermatozoa. Spermatozoa isolated from undisturbed rats were treated ex vivo, for 6 h, either with adrenergic receptors (ADRs) agonists (adrenaline, isoproterenol) or/and antagonists (ɑ1-ADRs antagonists prazosin; β-ADRs antagonists propranolol) or glucocorticoid receptors (GRs) agonist (hydrocortisone) or/and antagonist (RU486). The RNA was used for analyses of the transcriptional profile of mitochondrial fission markers (**a**, **b**). Data bars are mean ± SEM values of three independent ex vivo experiments involving six rats per experiment (eighteen in total). Statistical significance was set at level *p* < 0.05: * vs. basal group.

Since process of mitochondrial fission is required for homeostasis of mitochondrial function and network^43,44,47^, the main markers (*Fis1,* *Drp1*) of mitofission were followed in spermatozoa (Fig. 5a,b). Results showed that the level of *Fis1* transcript in spermatozoa significantly increased (1.6-fold) by hydrocortisone and this effect was completely abolished in presence of GRs-specific antagonist RU486. *Drp1* transcription was increased by both stress mimetics. Adrenaline significantly increased (2.1-fold) *Drp1* transcription and this effect was completely abolished with both ɑ1-ADRs-antagonist and β-ADRs-antagonist, suggesting the involvement of both types of ADRs. Moreover, combination of adrenaline with ɑ1-ADRs-antagonist prazosin not only abolished the effect of adrenaline, but also caused significant reduction (4.7-fold) comparing to control, suggesting the most prominent effect of ɑ1-ADRs. The agonist of GRs, hydrocortisone, significantly increased (1.8-fold) *Drp1* transcript and this increase was persistent (2.1-fold) even in presence of combination of agonist with GR-specific antagonist RU486.

### The stress hormones change the transcriptional profile of mitochondrial autophagy markers in spermatozoa

**Figure 6:**
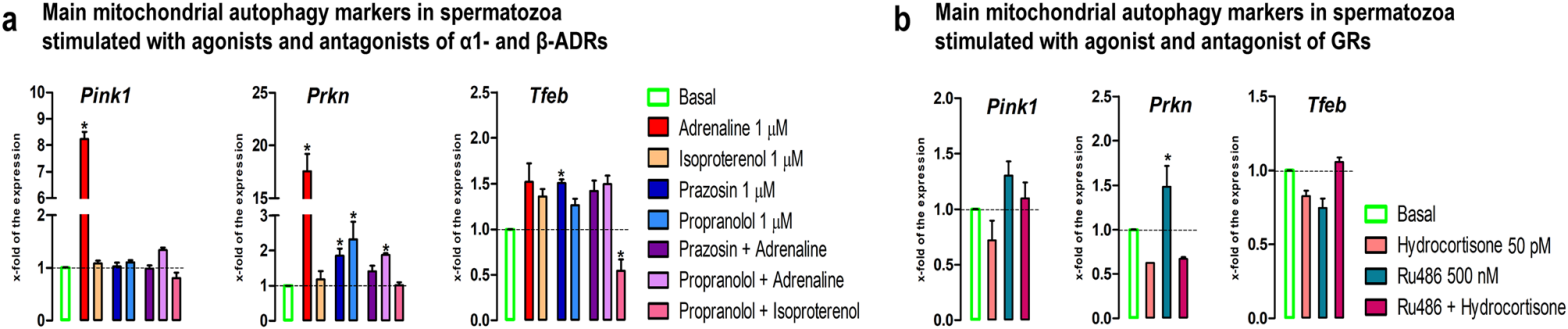
The stress hormones change the transcriptional profile of mitochondrial autophagy markers in spermatozoa. Spermatozoa isolated from undisturbed rats were treated ex vivo, for 6 h, either with adrenergic receptors (ADRs) agonists (adrenaline, isoproterenol) or/and antagonists (ɑ1-ADRs antagonists prazosin; β-ADRs antagonists propranolol) or glucocorticoid receptors (GRs) agonist (hydrocortisone) or/and antagonist (RU486). The RNA was used for analyses of the transcriptional profile of mitochondrial autophagy markers (**a**, **b**). Data bars are mean ± SEM values of three independent ex vivo experiments involving six rats per experiment (eighteen in total). Statistical significance was set at level *p* < 0.05: * vs. basal group.

Besides all previously mentioned processes of mitochondrial dynamics, mitophagy is also crucial for homeostasis of mitochondrial function and network^43,44,47^. Accordingly, the main markers (*Pink1,* *Prkn,* *Tfeb*) of mitophagy were followed in spermatozoa (Fig. 6a,b). Results showed that adrenaline significantly increased (8.2-fold) the level of *Pink1* transcript and this effect was completely abolished with both, ɑ1-ADRs-antagonist and β-ADRs-antagonist, suggesting the involvement of both types of ADRs. More prominent increase (17.6-fold) by adrenaline treatment was observed on *Prkn* transcript, but this effect was completely abolished only with ɑ1-ADRs-antagonist, while β-ADRs-antagonist just diminished the effect of adrenaline, suggesting that ɑ1-ADRs are more involved in adrenaline-mediated stimulation. Moreover, blockade of ɑ1-ADRs and β-ADRs significantly increased (prazosin—> 1.9-fold, propranolol—> 2.3-fold) *Prkn* transcript. The adrenaline abolished effect of prazosin, but diminished effect of propranolol (1.9-fold vs. 2.3-fold), while β-ADRs-agonist-isoproterenol completely abolished effect of propranolol. GRs-antagonist RU486 significantly increased (1.5-fold) *Prkn* transcript and this effect was completely abolished with GRs-agonist-hydrocortisone. The level of *Tfeb* transcript significantly increased with ɑ1-ADRs-antagonist-prazosine (1.5-fold) and this effect was abolished in combination with adrenaline, while combination of β-ADRs antagonist and agonist (propranolol + isoproterenol) significantly decreased (1.9-fold), suggesting the complex regulation of *Tfeb* transcription.

Since the effects of adrenaline were more prominent than the effects of GRs-agonist, the transcriptional profile of the main markers of adrenergic signaling was followed.

### The adrenaline disturbs the transcriptional profile of adrenergic receptors and adrenergic receptor kinases and β-ADRs signaling in spermatozoa

**Figure 7:**
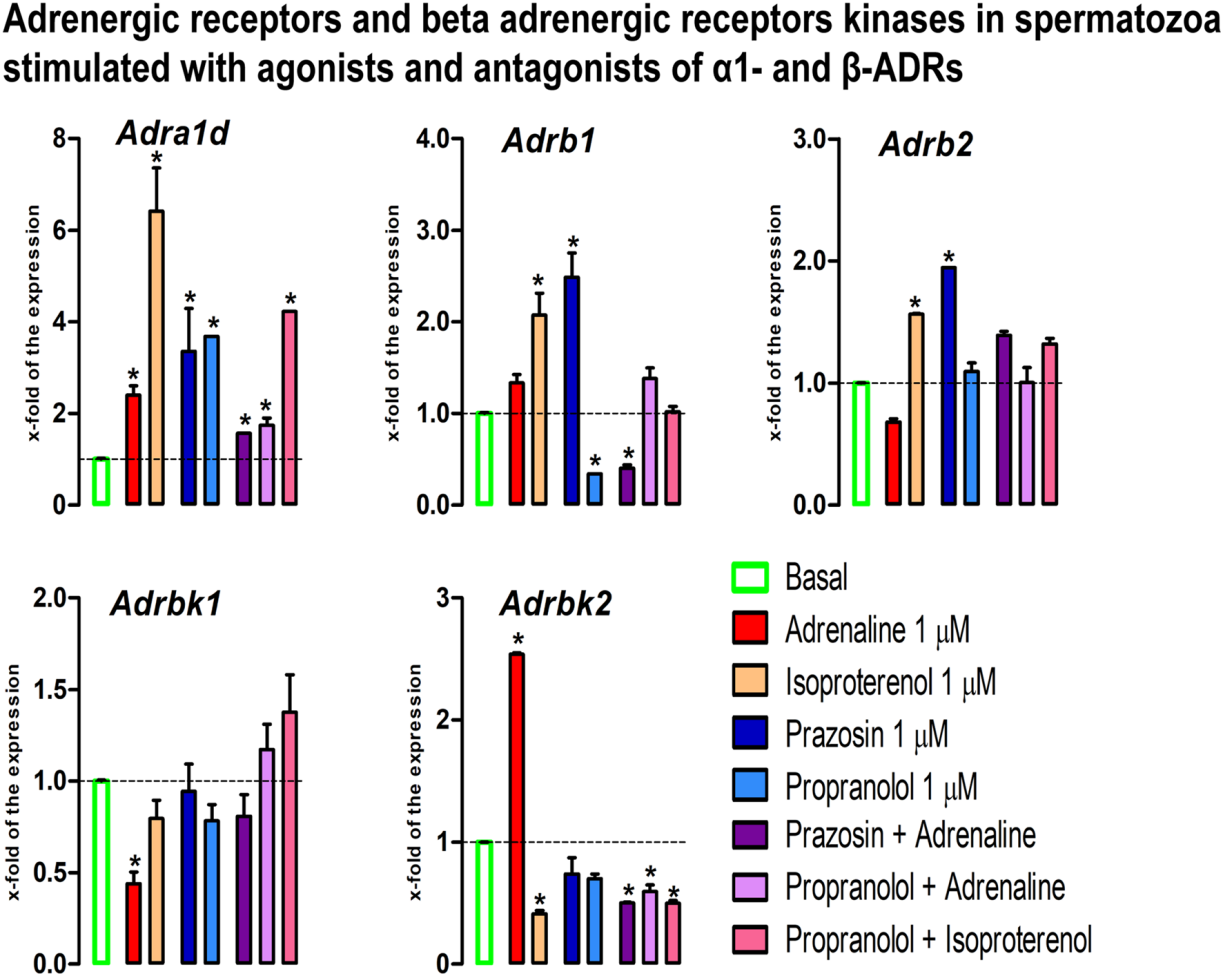
The adrenaline disturbs the transcriptional profile of adrenergic receptors and adrenergic receptor kinases in spermatozoa. Spermatozoa isolated from undisturbed rats were treated ex vivo, for 6 h, either with adrenergic receptors (ADRs) agonists (adrenaline, isoproterenol) or/and antagonists (ɑ1-ADRs antagonists prazosin; β-ADRs antagonists propranolol). The RNA was used for analyses of the transcriptional profile of ADRs and β-ADRs kinases. Data bars are mean ± SEM values of three independent ex vivo experiments involving six rats per experiment (eighteen in total). Statistical significance was set at level *p* < 0.05: * vs. basal group.

The results showed that transcriptional profiles of ADRs and their kinases are under the significant influence of adrenergic signaling (Fig. 7). The level of the *Adra1d* transcript significantly increased in spermatozoa incubated with adrenaline (2.4-fold), isoproterenol (6.4-fold), prazosin (3.4-fold), propranolol (3.7-fold), prazosin + adrenaline (1.6-fold), propranolol + adrenaline (1.7-fold), propranolol + isoproterenol (4.2-fold). In the same samples, the *Adrb1* transcript level increased by isoproterenol (2.1-fold) and prazosin (2.5-fold), while decreased by propranolol (2.9-fold) and combination-prazosin + adrenaline (2.5-fold). Besides, the level of transcript for the most abundantly expressed ADRs, *Adrb2*, significantly increased by isoproterenol (1.6-fold) and prazosin (1.9-fold). The transcriptional profiles of kinases associated with ADRs signaling were also disturbed. The level of *Adrbk1* significantly decreased (2.3-fold) in spermatozoa incubated with adrenaline and this effect was completely abolished with both ɑ1-ADRs-antagonist and β-ADRs-antagonist, suggesting the involvement of both types of ADRs. The *Adrbk2* transcript increased by adrenaline (2.5-fold) and this effect was not only abolished by ɑ1-ADRs-antagonist/β-ADRs-antagonist but lead to decrease (prazosin + adrenaline: twofold, propranolol + adrenaline: 1.7-fold). Oppositely, the level of *Adrbk2* transcript significantly decreased (2.4-fold) in spermatozoa incubated with β-ADRs-agonist-isoproterenol and this decrease-effect was persistent (twofold) even in the presence of β-ADRs-antagonist (propranolol + isoproterenol).

## Discussion

There is so far no single all-encompassing biomarker of reproductive capacity in men and/or biomarkers for male reproductive health hazards. Our results are the first to show the importance of mitochondrial biogenesis and fusion/architecture markers in spermatozoa since the transcriptional profile of eleven-out-of-fourteen were disturbed by manipulation of stress-hormones-signaling. The stress-hormones-trigger changes in the profile of molecules responsible for mitochondrial biogenesis and fusion/architecture in spermatozoa and these changes do not only correlate with spermatozoa functionality, but also represents an adaptive mechanism essential for spermatozoa functionality, being both events depend on the same regulators. With this transcriptional signaling scenario, the spermatozoa may be trying to preserve the basic mitochondrial and self-activity. Several lines of evidence prove that the stress alters the signaling pathways and molecules responsible for mitochondrial biogenesis and fusion/architecture in spermatozoa with consequences for the function. (1) Repeated psychophysical stress by immobilization reduced the number of spermatozoa. (2) Both types of stress, acute and repeated, significantly reduced spermatozoa functionality. (3) The ex vivo application of stress hormones adrenaline and hydrocortisone mimicked the effect of stress on spermatozoa functionality. (4) Stress hormones significantly disturb the transcriptional profile of the sixteen out of nineteen markers of mitochondrial biogenesis, mitofusion/mitoarchitecture, mitofission and mitophagy and some of the effects are specific for one type of adrenergic receptor, while some of the effects are regulated by several types of the adrenergic receptors. (5) The manipulation of adrenergic signaling in spermatozoa by using the agonists or/and antagonists revealed the complex regulation of the transcription of main adrenergic receptors and adrenergic receptors kinases on spermatozoa.

Our results are in line with results showing that chronic intermittent stress irreversible decrease sperm count^5^, significantly enhanced apoptosis in germ cells and decreased the number of spermatogenic cells^52^, significantly decreased sperm counts, sperm motility, sperm viability^53^ and sperm quality^54^ in male rats. In humans, it has been shown that stress related to recent death of a close family member was associated with a reduction in percentage of progressively motile sperm^55^. Besides, secondary infertility was significantly higher in patients with post-traumatic stress disorder^56^. Our results showing the inhibitory role of stress hormones on sperm functionality are supported by the data presenting stress-induced-GRs-signaling-mediate spermatogenesis impairment^57^ as well as reduced testosterone and sperm motility in high and moderate male runners^58^. Oppositely, in α1-ADRs-knockout-male-mice^22^ fertility and spermatogenesis are altered, suggesting the important and complex involvement of adrenergic signaling in spermatogenesis and fertility.

Given the crucial role of mitochondria in cell physiology, it is obvious that these organelles are among the first responders to various stressors challenging homeostasis of the cell and organism^8,9^. Our results are the first to show the importance of mitochondrial network dynamics markers in spermatozoa since the transcriptional profile of sixteen-out-of-ninteen were disturbed by manipulation of stress-hormones-signaling. The level of *Ppargc1a* significantly decreased in spermatozoa incubated with isoproterenol, β-ADRs-agonist, but also in those incubated with propranolol, β-ADRs-antagonist, and both effects were abolished by the combination (propranolol + isoproterenol), suggesting the involvement of β-ADRs. Oppositely, the transcriptions of *Ppargc1b,* *Cox4i1,* *Ucp1* are significantly increased by adrenaline and these effects were completely abolished with both ɑ1-ADRs-antagonist and β-ADRs-antagonist, suggesting the involvement of both types of ADRs. Increased levels of *Ppargc1a* and *Ucp3* in presence of ɑ1-ADRs-antagonist-prazosin could be explanation for positive effects of alpha-blockers in oligoizoospermic man since two placebo-controlled, double-blind clinical studies concluded that alpha-blockers are a useful drug in the treatment of idiopathic moderate oligozoospermia^59,60^. In addition, fertility and spermatogenesis are altered in α1-ADRs-knockout-male-mice^22^. Our results show that the level of transcript for *Tfam* decreases by β-ADRs-agonist-isoproterenol and β-ADRs-antagonist-propranolol abolish this effect. This is in line with findings that TFAM is associated with the reduction in mtDNA content of human sperm^34^ and that TFAM gene expression positively correlate with abnormal forms, sperm DNA fragmentation and mtDNA copy number^35,36^. Our results showing that combination β-ADRs-antagonist-propranolol + β-ADRs-agonist-isoproterenol significantly decreased the level of *Ucp2* suggest the positive involvement of β-ADRs in *Ucp2* regulation and could be possible explanation for findings that UCP2 mitigates the loss of human spermatozoa motility^38^. The effects of β-ADRs-agonist-isoproterenol were not always in parallel with the effects of adrenaline mediated through β-ADRs and β-ADRs-antagonist-propranolol was not always diminished/abolished the effects of isoproterenol. The possible explanation could be the dose of isoproterenol, since two published papers presented data obtained using lower concentration (0.2 μM) of isoproterenol^61^ and stating that isoproterenol (0.2 μM) speed the flagelar beat of mammalian sperm by a non-receptor-mediated mechanism^62^.

The transcripts for main markers (*Mfn1,Mfn2,Opa1*) of mitochondrial fusion/architectures, important for homeostasis of mitochondrial function and network^43,44,47^, were dramatically increased by adrenaline (*Mfn1*—3.4-fold; *Mfn2*—7.5-fold; *Opa1*—2.7-fold) and these effects were completely abolished with both ɑ1-ADRs-antagonist and β-ADRs-antagonist, suggesting the involvement of both types of ADRs. Also, the level of *Mfn1* transcript significantly decreased (2.1-fold) in spermatozoa incubated with combination-prazosin + adrenaline, suggesting that ɑ1-ADRs could mediate more stimulatory effects. These results may explain relation of the expression level of MFN2 to motility and cryoprotective potentials of human sperm^39^. The increased expression of transcripts for all mitochondrial fusion/architectures markers could be also adaptive mechanism to survive the disturbed homeostasis. Namely, process of mitofusion provide environment for exchange of biomolecules between mitochondria, while condensed cristae are markers of higher production of ATP. Moreover, most prominent increase (7.5-fold) of *Mfn2* transcript could lead to increase in MFN2 protein level and could provide stronger connection of mitochondria with endoplasmic reticulum leading to increase in exchange of Ca^2+^, the second messenger critical for all mechanisms crucial for the spermatozoa functionality. In parallel with the changes in the transcription profiles of mitofusion markers, similar effects were observed on the transcriptional profiles of mitofission markers.The level of *Fis1* transcript increased (1.6-fold) by hydrocortisone was completely abolished in presence of GRs-specific antagonist RU486, but was not affected with adrenergic signaling, suggesting the sole involvement of GRs-signaling in the transcriptional regulation of *Fis1* gene. On the other hand, *Drp1* transcription increased with both stress mimetics. The adrenaline effect was completely abolished in the presence of the blocker, while effect of GRs-agonist was persistent even in the presence of GRs-blocker, suggesting the either sole involvement of ADRs, or that maybe concentration of GRs-blocker was not appropriate.

The transcriptional profiles of main mitophagy markers (*Pink1,* *Prkn*) also dramatically increased (8.2-fold for *Pink1*; 17.6-fold for *Prkn*). The effect of adrenaline on *Pink1* transcript was completely abolished with both, ɑ1-ADRs-antagonist and β-ADRs-antagonist, suggesting the involvement of both types of ADRs. More prominent increase on *Prkn* transcript was completely abolished only with ɑ1-ADRs-antagonist, while β-ADRs-antagonist just diminished the effect of adrenaline, suggesting that ɑ1-ADRs are more involved in adrenaline-mediated stimulation. Moreover, blockers of ɑ1-ADRs and β-ADRs significantly increased (prazosin-> 1.9-fold, propranolol-> 2.3-fold) *Prkn* transcript. The adrenaline abolished effect of prazosin, but diminished effect of propranolol (1.9-fold vs. 2.3-fold), while β-ADRs-agonist-isoproterenol completely abolished effect of propranolol, suggesting complex and specific regulation of *Prkn* transcription by adrenergic signaling. Oppositely, GRs-signaling is negatively involved in regulation of *Prkn* transcription since GRs-antagonist-dependent increase (1.5-fold) of *Prkn* transcript was completely abolished with GRs-agonist-hydrocortisone. The level of *Tfeb* transcript significantly increased with ɑ1-ADRs-antagonist-prazosine (1.5-fold) and this effect was abolished in combination with adrenaline, while combination of β-ADRs antagonist and agonist (propranolol + isoproterenol) significantly decreased (1.9-fold), suggesting the complex regulation of *Tfeb* transcription.

Accordingly, manipulation of stress-signaling in spermatozoa by using the agonists or/and antagonists of ADRs or GRs reveals that most of these effects are mediated through ɑ1-ADRs and/or β-ADRs.

A final important insight from our study is that the adrenergic signaling disturbs transcriptional profile of ADRs and their kinases and that regulation of their transcription is intriguing and complex involving both ɑ1-ADRs and β1-ADRs. It is difficult to provide precise mechanism since it is very well known that ADRs communicate with each other in regulation of their expression in health and diseases^20^. However, it is clear that the transcription of *Adrbk2* is significantly increased by adrenaline and this effect was completely abolished with both ɑ1-ADRs-antagonist and β-ADRs-antagonist, suggesting the involvement of both types of ADRs. The physiological significance is obvious since it has been shown that mammalian spermatozoa β-ADRs stimulate cAMP production by membrane-associated adenylyl cyclases^63^.

Why all the above mention is important? As was mentioned before, although our reality is a significant increase of unexplained cases of male infertility in humans, especially of infertile males in the peak of the reproductive period (under age 30), the mechanisms are unknown^1,2^. The facts that “life at the top” and alpha males exhibited much higher stress hormone levels than second-ranking (beta) males^1,2^ and that the semen quality and male fertility are important not only as of the fundamental marker of reproductive health but also as the fundamental biomarkers of overall health, ask urgent reaction^3^. However, the exact nature of these associations remains somewhat unclear, although hypothesized mechanisms include genetic, developmental, and lifestyle-based factors^3^. We believe that our results provide a completely new view on spermatozoa energetic homeostasis and testing of spermatozoa functionality and (in)fertility and that in the future could serve as Mito-Fet-Sperm-Signature diagnostic test.

## Conclusion

**Figure 8:**
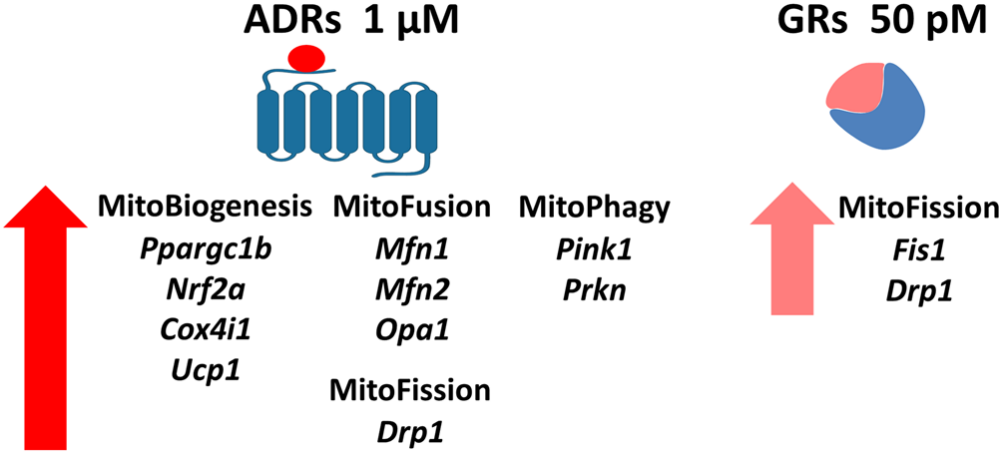
The stress mimetics disturb (mostly increase) sixteen out of nineteen mitochondrial dynamics markers in spermatozoa with adrenergic signaling being more effective, suggesting the importance of these spermatozoa markers in response on high energy demand during stress. The all effects of adrenaline are stimulatory and most of them are completely abolish or at least diminish with blockade of ɑ1-ADRs and/or β-ADRs, suggesting the adrenergic-mediated increase of mitochondrial network dynamics as adaptation and proper response on high energy demand. The specific effect of GRs was observed on increased level of *Fis1* transcript.

Stress-hormones-trigger changes in the transcriptional profile of mitochondrial dynamics markers, as well as adrenergic receptors and adrenergic receptors kinases are important molecular markers of spermatozoa functionality representing an adaptive mechanism regulated by stress signaling and does not only correlate-with but also are essential for spermatozoa functionality, being all events depend on the same regulators. The stress mimetics disturb (mostly increase) sixteen out of nineteen mitochondrial dynamics markers in spermatozoa (Fig. 8) with adrenergic signaling being more effective, suggesting the importance of these spermatozoa markers in response on high energy demand during stress. Accordingly, the above mentioned molecular markers can be used as a test for spermatozoa functionality and for a better understanding of the correlation between stress as well as any other life-style-environmental-one-health-factors and male (in)fertility.

## Materials and methods

Most of the methods used in the present study were previously reported by our group in more detail (for all references please see^16,17,64^ as well as in Supplemental Material and Methods, and are outlined briefly here.

### Animals and ethical statement

Adult male *Wistar* rats were bred and raised in controlled environmental conditions with food and water ad libitum in the accredited Animal Facility (Faculty of Sciences, University of Novi Sad). All experimental protocols were approved (statement no. 01-201/3) by the local Ethical Committee on Animal Care and Use of the University of Novi Sad and were performed in accordance with the rules of National Council for Animal Welfare and the National Law for Animal Welfare (copyright March 2009), following the NRC publication Guide for the Care and Use of Laboratory Animals and NIH Guide for the Care and Use of Laboratory Animals. Experiments were performed in the Laboratories LaRES and ChronAge (https://wwwold.dbe.pmf.uns.ac.rs/en/nauka-eng/lares).

### In vivo model of psychophysical stress by immobilization

Psychophysical stress by immobilization (IMO) was performed in the morning (from 07^00^ to 10^00^ h) by the method previously described^12,16,17^. Briefly, rats were divided into the following groups: Control—freely moving (unstressed) rats; 1 × 3hIMO—rats subjected to IMO once, for 3 h; 10 × 3hIMO—rats subjected to repeat IMO of 3 h for 10 consecutive days. At the end of the IMO period, all the animals were quickly decapitated without anesthesia and trunk blood was collected. Serum samples were collected and assayed for androgens (testosterone + dihydrotestosterone; T + DHT), adrenaline and corticosterone (CORT) levels. The experiments were repeated four times. The numbers of animals per each group are presented on the top of the bars (please see Figs. 1 and 2).

### Hormones measurement in serum

The levels of hormones in serum samples were measured in duplicate in one assay. **Androgens** levels were referred to as T + DHT since anti-testosterone serum №250 showed 100% cross-reactivity with DHT (assay sensitivity: 6 pg per tube; intra-assay coefficient of variation 5–8%). **Adrenaline** levels were measured using the adrenaline research ELISA Kit (www.ldn.de) with the standard range of 0.45–45 ng/ml and detection limit of 3.9 pg/ml. **Corticosterone** levels were measured by the corticosterone EIA Kit (www.caymanchem.com) with 30 pg/ml as the lowest standard significantly different from blank.

### Isolation of spermatozoa

Spermatozoa were isolated from caudal epididymides following the WHO laboratory manual (https://www.who.int/reproductivehealth/publications/infertility/9789241547789/en/*)* with modifications for rat spermatozoa isolation. Caudal epididymides were quickly removed, placed in a petri dish containing the medium for isolation and preservation of spermatozoa (1% M199 in HBSS with 20 mM HEPES buffer and 5% BSA), finely punctuated with needle and incubated for 10 min (37 °C). After the incubation, released spermatozoa were collected, centrifuged 5 min/700*xg*, and resuspended in the appropriate medium. The numbers of isolated spermatozoa were calculated using a Makler counting chamber.

### Ex vivo treatment of spermatozoa isolated from undisturbed rats

The effects of stress hormones on spermatozoa functionality (% acrosome-reacted-spermatozoa) were followed after incubation of spermatozoa with adrenaline (10 μM, 100 μM) or hydrocortisone (50 pM, 500 pM) for 30 min (37 °C). The transcriptional profiles were followed after incubation of spermatozoa (1 × 10^6^ in DMEM/F12 medium) for 6 h (37 °C) with adrenaline (1 μM) alone or in combination with adrenergic receptors (ADRs) antagonists, ɑ1-antagonist prazosin (1 μM) and β-antagonist propranolol (1 μM). For the stimulation of only β-ADRs, spermatozoa were incubated with β-agonist isoproterenol (1 μM) alone or in combination with propranolol (1 μM). To investigate the effect of agonist and/or antagonist of glucocorticoid receptors (GRs), spermatozoa were incubated with hydrocortisone (50 pM) and/or antagonist RU486 (500 nM). After the incubation period, spermatozoa were centrifuged 7 min/1000*×g* and stored at − 80 °C until RNA isolation. Four replicates of each group were used and all ex vivo experiments were repeated three times.

### Capacitation and acrosome reaction of spermatozoa

To determine the functionality of the spermatozoa after the in vivo and ex vivo experiments approximately 1.5 × 10^5^ spermatozoa were incubated in Whitten’s Media supplemented with the 10 mg/ml BSA and 20 mM NaHCO_3_, for 1 h (37 °C). After the incubation, capacitated spermatozoa were treated with progesterone (15 μM), to activate acrosome reaction, or incubated without progesterone, for 30 min (37 °C). Following the stimulation of acrosome reaction, spermatozoa were fixed with fixation solution for 20 min (RT), and centrifuged for 1 min/12000*xg*. Spermatozoa in the pellet were washed with 100 mM ammonium acetate, pH 9. Smears of fixed spermatozoa on microscopic slides were air-dried and stained with a solution containing 0.04% Coomassie Blue for 5 min (RT), rinsed with distilled water and air-dried. Stained smears were analyzed, and up to 100 spermatozoa/slide counted to determine the acrosomal status. Blue staining in the acrosomal region of the head indicated intact acrosome, whereas spermatozoa without blue staining in the acrosomal region were considered acrosome-reacted. Data are presented as the percentage of acrosome-reacted spermatozoa ± SEM.

### RNA isolation and cDNA synthesis

Total RNA was isolated using GenElute™ Mammalian Total RNA Miniprep Kit (www.sigmaaldrich.com) following the DNase I (RNase-free) treatment (www.neb.com) according to the manufacturer’s protocols. First-strand cDNA was synthesized using the High Capacity Kit following the manufacturer’s instructions (www.thermofisher.com). Quality of RNA and DNA integrity was checked using control primers for *Gapdh*.

### Real-time PCR and relative quantification

The quantification of relative gene expression was done by real-time PCR (RQ-PCR) using SYBR®Green-based chemistry (www.thermofisher.com) in the presence of specific primers (please see Supplemental Tables 1, 2 and 3). The transcription of *Gapdh* was measured, and used to correct the variations in cDNA content between the samples. Relative quantification of each gene was performed in duplicate, three times for each sample of three independent ex vivo experiments.

### Statistical analysis

The results represent group means ± SEM values of the individual variation from three to four independent experiments. Results from each experiment were analyzed by Mann–Whitney's unpaired nonparametric two-tailed test (for two-point data experiments), or by one-way ANOVA for group comparison, followed by Student–Newman–Keuls multiple range test. All the statistical analyses were done using GraphPad Prism 5 Software, and *p* value < 0.05 was considered to be statistically significant.

## Supplementary information

Supplementary Information.

## Data Availability

All relevant data are available from the corresponding author on request. Further information and requests for data, resources and reagents should be directed to and will be fulfilled by the corresponding author, Silvana Andric (silvana.andric@dbe.uns.ac.rs).
