# Supplementary material for: Reduced spermatozoa functionality during stress is the consequence of adrenergic-mediated disturbance of mitochondrial dynamics markers
Source: Sci Rep. 2020 Oct 8;10:16813. doi: 10.1038/s41598-020-73630-y (PMC7544694; doi:10.1038/s41598-020-73630-y)
Supplement: Supplementary file 1 — Supplementary Information. [file 41598_2020_73630_MOESM1_ESM.pdf]

TITLE PAGE

Running title
Adrenaline disturbs sperm mito-markers

Manuscript Title
Reduced spermatozoa functionality during stress is the consequence of adrenergic-mediated disturbance of mitochondrial dynamics markers

Authors
Isidora M Starovlah, Sava M Radovic Pletikosic, Tatjana S Kostic, Silvana A Andric *

SUPPLEMENTARY RESULTS

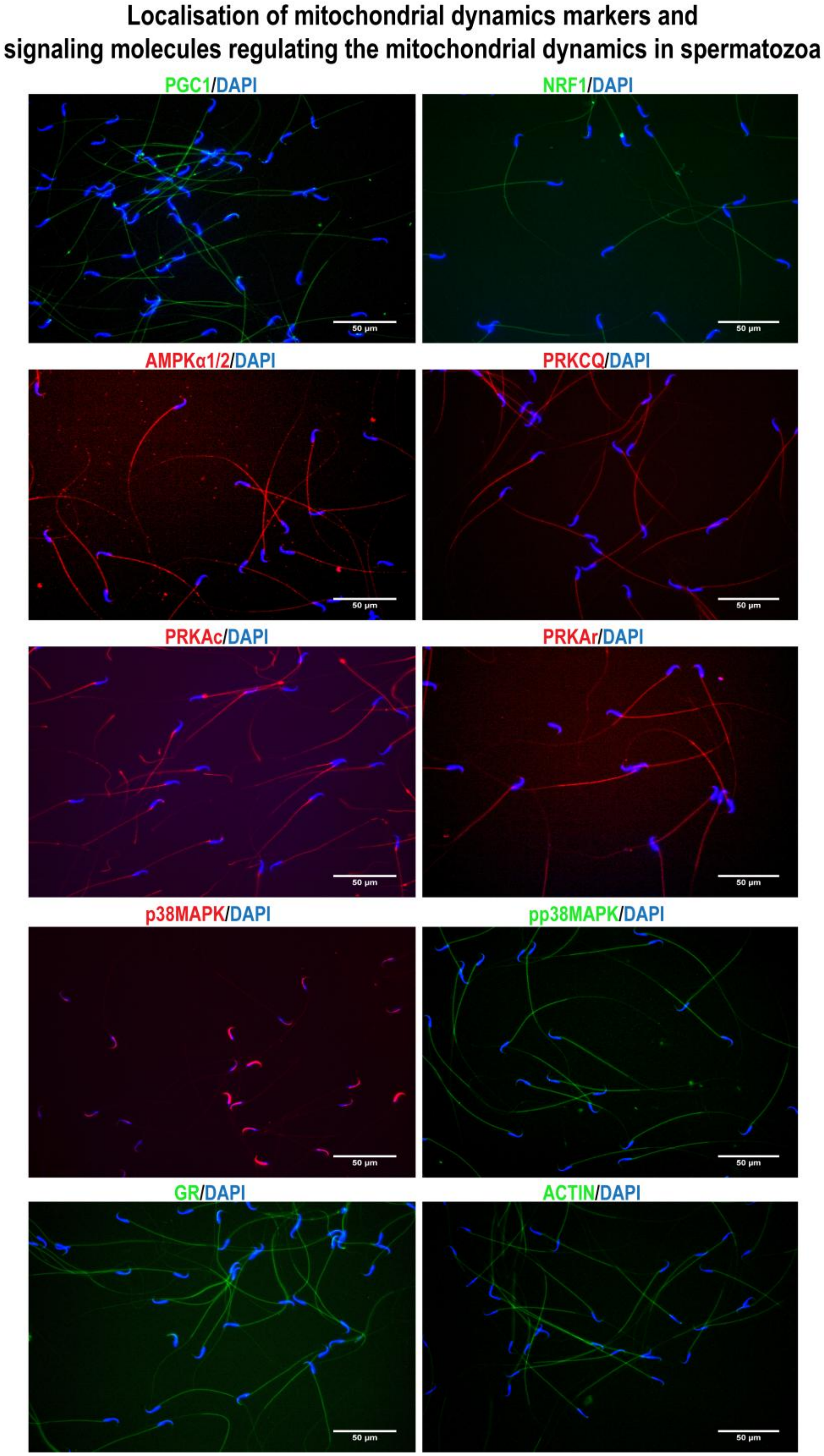

Supplementary figure S1. **Localization of the main mitochondrial dynamic markers and signaling molecules regulating mitochondrial dynamic using immunofluorescence staining of adult spermatozoa.**
To identify the presence and localization of the main mitochondrial dynamic markers and signaling proteins regulating mitochondrial dynamic, immunofluorescence was conducted. Fixed spermatozoa were permeabilized, blocked and incubated with primary antibodies against PGC1, NRF1, AMPKα1/2, PRKCQ, PRKAc, PRKAr, p38MAPK, pp38MAPK, GR and ACTIN. Secondary immunofluorescent antibodies Alexa Fluor 555 and Alexa Fluor 488, with Fluoroshield Mounting Medium with DAPI were used for the visualisation. Scale bar 50 µm.

SUPPLEMENTARY MATERIALS AND METHODS

Key resources table

Resource or reagent	Source	Identifier
**Experimental model and biological samples**		
Wistar rat	LaRES and ChronAge Laboratories (DBE, Faculty of Sciences, University of Novi Sad)	http://wwwold.dbe.pmf.uns.ac.rs/en/nauka-eng/lares
Primary culture of spermatozoa	Three to four months-old male rats	NA
Serum	Three to four months-old male rats	NA
**Commercial Reagents/Assays**		
Adrenaline research ELISA Kit	Labor Diagnostika Nord	https://ldn.de
Corticosterone EIA Kit	Cayman	https://www.caymanchem.com
GenElute™ Mammalian Total RNA Miniprep Kit	Sigma Aldrich	https://www.sigmaaldrich.com
DNase I (RNase-free) treatment	New England Biolabs	https://international.neb.com
High Capacity Kit for cDNA	Applied Biosystems/Thermo Fisher Scientific	https://www.thermofisher.com
Power SYBR® Green PCR Master Mix	Applied Biosystems/Thermo Fisher Scientific	https://www.thermofisher.com
**Primers**		
Supplemental tables S1 to S5	This paper	www.ncbi.nlm.nih.gov/sites/entrez
**Software**		
GraphPad Prism 5 Software	GraphPad Prism	https://www.graphpad.com/scientific-software/prism

Animals and ethical statement

Three to four-months-old (260-350 g) male *Wistar* rats, bred and raised in the Animal Facility of the Faculty of Sciences, University of Novi Sad, Serbia were used for experiments. Animals were raised in controlled environmental conditions (22 ± 2 °C; 14 hours light and 10 hours dark cycle, lights on at 07^{00} h) with food and water *ad libitum*. All experimental protocols were approved (statement no. 01-201/3) by the local Ethical Committee on Animal Care and Use of the University of Novi Sad and were performed in accordance with the rules of National Council for Animal Welfare and the National Law for Animal Welfare (copyright March 2009), and following the National Research Council publication Guide for the Care and Use of Laboratory Animals (copyright 1996, National Academy of Sciences, Washington DC)

and NIH Guide for the Care and Use of Laboratory Animals (NIH Publications No. 80 23, revised 1996, 7th edition; *www.nap.edu/readingroom/books/labrats*). All experiments adhere to APS's Guiding Principles in the Care and Use of Vertebrate Animals in Research and Training and were carried out in the Laboratory for Reproductive Endocrinology and Signaling and Laboratory for Chronobiology and Aging, Faculty of Sciences at University of Novi Sad (*http://wwwold.dbe.pmf.uns.ac.rs/en/nauka-eng/lares*).

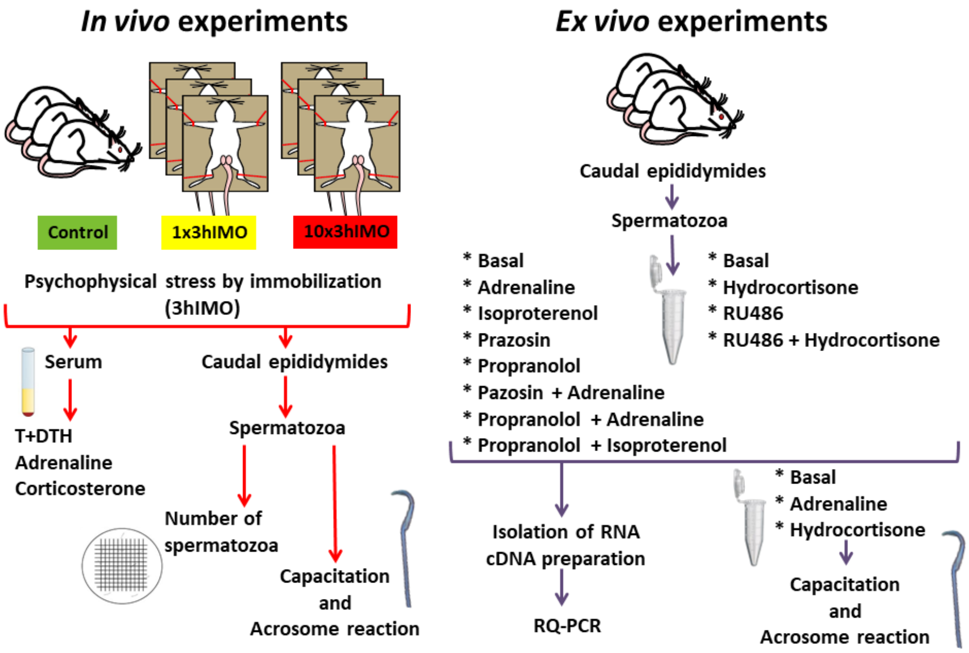

Supplemental scheme S1. **Experimental design used for research of functionality (% acrosome reaction) and transcriptional profile of mitochondrial biogenesis and fusion/architecture markers in spermatozoa.**

In vivo model of psychophysical stress by immobilization

Psychophysical stress by immobilization (IMO) was performed in the morning (from 07^{00} h to 10^{00} h) by the method previously described[12,16.17] . Briefly, 3hIMO rats were bound in a supine position to a wooden board by fixing the rats' limbs using thread, while the head motion was not limited. Rats were divided into the following groups consisting of four to six animals each: Control group – freely moving (unstressed) rats; 1x3hIMO – rats subjected to IMO once, for 3 hours; 10x3hIMO – rats subjected to repeat IMO of 3 hours for 10 consecutive days. At the end of IMO period control and the 3hIMO animals were quickly decapitated without anesthesia and trunk blood was collected. Individual serum samples were stored at -80 °C until they were assayed for androgens (testosterone + dihydrotestosterone; T+DHT), adrenaline and corticosterone (CORT) levels. The experiments were repeated four times. For the number of the animals please see the numbers in the brackets above the bars in the Figures.

Hormones measurement in serum

Level of androgens in serum was referred to as T+DHT since the anti-testosterone serum №250 showed 100% cross-reactivity with DHT (for references please see Gak/Radovic et al. 2015, Starovlah et al. 2017). All samples were measured in duplicate in one assay (sensitivity: 6 pg per tube; intra-assay coefficient of variation 5-8%). For serum adrenaline levels, all samples were measured in duplicate, using the adrenaline research ELISA Kit (*www.ldn.de*) with the standard range of 0.45-45 ng/ml and detection limit of 3.9 pg/ml. Serum corticosterone (CORT) levels in all samples were measured in duplicate in one assay by the corticosterone EIA Kit (*www.caymanchem.com*) with 30 pg/ml as the lowest standard significantly different from blank.

Isolation of spermatozoa

Spermatozoa were isolated from caudal epididymides following the WHO laboratory manual (*https://www.who.int/reproductivehealth/publications/infertility/9789241547789/en/*) with modifications for rat spermatozoa isolation. Caudal epididymides were quickly removed, placed on filter paper and the surrounding adipose tissue removed. The epididymides were placed in a petri dish containing 4 ml of the medium for isolation and preservation of spermatozoa (1% M199 in HBSS with 20 mM HEPES buffer and 5% BSA) and finely punctuated with 25G needle to enable spermatozoa to be released into the medium, after what they were incubated at 37 °C for 10 minutes. After the incubation period, released spermatozoa in the medium were collected, centrifuged 5 minutes at 700*xg* at room temperature, the supernatant was removed and the pellet resuspended in the appropriate medium, depending on the treatment performed. Concentrations of isolated spermatozoa were calculated using a Makler counting chamber (Sefi-Medical Instruments, ltd).

Immunofluorescence

Localization of the main mitochondrial dynamic markers and signaling proteins regulating mitochondrial dynamic was determined using the immunofluorescence method previously described (Matamoros-Volante et al. 2018), with some modifications. Five undisturbed adult rats were used for this experiment. After the isolation procedure, the concentrations of isolated spermatozoa were calculated and spermatozoa were diluted in WH medium, and 90x106 spermatozoa were used for immunofluorescence procedure (12 ml per 50 ml tube). Spermatozoa were incubated for 1 hour at 37 °C (5% CO2). After the incubation period, spermatozoa were centrifuged 5 min/800*xg*, supernatant discarded while spermatozoa in the pellet were washed with 1xPBS solution and centrifuged 5 min/800*xg*. Spermatozoa were fixed in 2% (*v/v*) paraformaldehyde in 1xPBS for 20 minutes at room temperature. Spermatozoa were centrifuged at 5 min/800*xg*, washed with 1xPBS, centrifuged 5 min/800*xg* and resuspended in 2 ml of 0.05% (*v/v*) Triton X-1000 in 1xPBS. Cells were permeabilized for 15 minutes at room temperature and after the incubation period washed with 1xPBS and centrifuged 5 min/4000*xg*. Spermatozoa were blocked with 3% BSA in 1xPBS solution for 2 hours at room temperature. Pull of blocked spermatozoa were divided into 1.5 ml test tubes (300 µl per 1.5 ml test tube), and incubated with primary antibody overnight at 4 °C. Antibodies against PGC1 (sc-13067), NRF1 (sc-33771), AMPKα1/2 (sc-74461) and ACTIN (sc-1616) were provided from Santa Cruz Biotechnology Inc (*www.scbt.com*). Antibodies against PRKAc (#610980) and PRKAr (#p53620) were provided from BD Transduction Laboratories (*www.bdbiosciences.com*), while antibodies against, PRKCQ (#9377S), p38MAPK (#9212) and pp38MAPK (#9211S) were provided from Cell Signaling Technology (*www.cellsignal.com*). Antibody against GR (#MA1-510) was provided by Thermo Fisher Scientific (*https://www.thermofisher.com*). After the incubation with the primary antibody, samples were washed with 1xPBS solution and

centrifuged 5 min/4000*xg*. Secondary immunofluorescent antibodies Alexa Fluor 555 (Abcam, ab150078 or ab150114) and Alexa Fluor 488 (Abcam, ab150077 or ab150113) were added in the samples (300 µl per 1.5 ml test tube) and incubated in dark for 1 hour at room temperature. All the antibodies and dilution ratio used in this experiment are given in *Supplemental Table S6.* After the incubation in secondary antibody, samples were washed with 1xPBS solution, centrifuged 5 min/4000*xg,* the supernatant discarded and the pellet was resuspended in the last drop of the 1xPBS solution. Two smears of each sample were prepared on microscopic slides and coverslips were mounted on slides with Fluoroshield Mounting Medium with DAPI (Abcam, ab104139). Slides were analyzed using a Leica DMLB100T microscope (Leica, Germany) and photographed on a Leica MC 190 HD camera (Leica, Germany).

Supplemental Table S6. The characteristics of the antibodies.

Target	Name of Antibody	Antigen sequence	Manufacturer, catalog #	Mono- or polyclonal	Dil. used
**PGC1**	Epitope mapping at amino acids 1-300 mapping near the N-terminus of PGC-1 of human origin	PGC-1 (H-300)<br>Peroxisome proliferative activated receptor gamma coactivator 1	Santa Cruz Biotech. Inc<br>sc-13067<br>MW (PGC1) =90kDa	Rabbit polyclonal IgG	1:50
**NRF1**	Epitope mapping amino acids 204-503 mapping at the C-terminus of human origin	NRF-1 (H-300)<br>Nuclear respiratory factor 1	Santa Cruz Biotech. Inc<br>sc-33771<br>MW (NRF1) = 68 kDa	Rabbit polyclonal IgG	1:50
**AMPKα1/2**	Amino acids 251-550 mapping at the C-terminus of AMPK1α of human origin	AMPKα1/2 (D-6)<br>5'-AMP/activated protein kinase	Santa Cruz Biotech. Inc<br>sc-74461<br>MW (AMPKα1/2) = 63 kDa	Mouse monoclonal IgG2a	1:50
**PRKCQ**	Phospho-PKCθ (Thr538) Antibody	Phospho-PKCθ (Thr538)	Cell Signaling Technology<br>#9377S<br>MW (PRKCQ) = 79 kDa	Rabbit polyclonal	1:500
**PRKAc**	Generated from human PRKACα subunit	Anti-Protein Kinase A catalitic subunit antibody	BD Transduction Laboratories<br>#610980<br>MW (PRKAc) = 40 kDa	Mouse monoclonal IgG2b	1:500
**PRKAr**	Generated from human PKA [RIα] aa. 1-381	Anti-PKA [RIα]	BD Transduction Laboratories<br>#p53620<br>MW (PRKAr) = 49 kDa	Mouse monoclonal IgG1	1:500
**p38MAPK**	p38 MAP Kinase Antibody detects endogenous levels of total p38α, -β, -γ MAPK protein	P38 MAPK Antibody	Cell Signaling Technology<br>#9212<br>MW (p38MAPK) = 43 kDa	Rabbit polyclonal	1:500
**pp38MAPK**	Synthetic phosphopeptide corresponding to residues around Thr180/Tyr182 of human p38	Phospho-P38 MAP Kinase (Thr180/Tyr182) Antibody	Cell Signaling Technology<br>#9211S<br>MW (pp38MAPK) = 43 kDa	Rabbit polyclonal IgG	1:500
**GR**	Glucocorticoid receptor Monoclonal Antibody (BuGR2)	Glucocorticoid receptor (BuGR2)	Thermo Fisher Scientific<br>#MA1-510<br>MW (GR) = 97 kDa	Mouse monoclonal IgG2	1:500
**ACTIN**	Epitope mapping at the Cterminus of ACTIN of human origin	ACTIN (I-19)	Santa Cruz Biotechnology Inc<br>sc-1616<br>MW (ACTIN) = 43 kDa	Goat polyclonal IgG	1:50

Ex vivo treatment of spermatozoa isolated from undisturbed rats

To investigate the effect of the stress hormones on functionality and transcriptional profile of mitochondrial biogenesis and fusion/architecture markers in spermatozoa, undisturbed rats were used. After the isolation procedure, the concentrations of isolated spermatozoa were calculated and spermatozoa were diluted in DMEM/F12 medium, and $1x10^6$ spermatozoa were used for *ex vivo* treatment (300 µl per 1.5 ml test tube). Four to five replicates of each group were used in each experiment. To observe the involvement of adrenergic receptors (ADRs) on the transcriptional profile of mitochondrial biogenesis and fusion/architecture markers, spermatozoa were incubated with adrenaline (1

μM) alone or in combination with α1-ADRs antagonist prazosin (1 μM) or β-ADRs antagonist propranolol (1 μM). Also, for the stimulation of only β-adrenergic receptors, spermatozoa were incubated with isoproterenol (1 μM) alone or in combination with β-ADRs antagonist propranolol (1 μM). To investigate the effect of glucocorticoid receptors on the transcriptional profile of mitochondrial biogenesis and fusion/architecture markers, spermatozoa were incubated with hydrocortisone (50 pM) alone or in combination with glucocorticoid receptor antagonist RU486 (500 nM). In all the above-mentioned *ex vivo* experiments, spermatozoa were incubated on an orbital shaker for 6 hours at 37 °C (5% CO_2). After the incubation period, spermatozoa were centrifuged 7 min/1000*xg,* supernatant discarded while spermatozoa in the pellet were stored at -80 °C, before RNA isolation. Concerning the functionality, spermatozoa were treated only with stress hormones adrenaline (10 μM and 100 μM) and hydrocortisone (50 pM and 500 pM) for 30 minutes at 37 °C (5% CO_2). After the incubation period spermatozoa samples went through the procedure of capacitation and acrosome reaction. All *ex vivo* experiments were repeated three times and total of eighteen undisturbed rats were used.

Capacitation and acrosome reaction of spermatozoa

To determine the functionality of the spermatozoa after the *in vivo* and *ex vivo* experiments approximately $1.5x10^5$ spermatozoa in 50 µl of Whitten's Media (100 mM NaCl, 4.7 mM KCl, 1.2 mM KH_2PO_4, 1.2 mM $MgSO_4$, 5.5 mM glucose, 1 mM pyruvic acid and 4.8 mM lactic acid) were mixed with 350 µl WH+ media (Whitten's Media supplemented with the 10 mg/ml BSA (Bovine Serum Albumin) and 20 mM of $NaHCO_3$, to stimulate the capacitation) with a drop of mineral oil, for 1 hour at 37 °C (5% CO_2).
After the incubation period, 50 µl of the capacitated spermatozoa were transferred into the new tubes, one without the progesterone and one with 15 μM progesterone (PROG), with the drop of mineral oil, for 30 minutes at 37 °C (5% CO_2). The progesterone was added to activate the acrosome reaction, where tubes without PROG were present as the control of the acrosome reaction. For the fixation of spermatozoa after the acrosome reaction, 20 µl of the spermatozoa suspension from each tube were mixed with 100 µl of the fixation solution (20 mM Na_2HPO_4, 150 mM NaCl and 7.5% formaldehyde) and incubated for 20 minutes at room temperature. After the fixation, spermatozoa were centrifuged for 1 minute at 12000*xg*. The supernatant was discarded and spermatozoa washed with 100 mM ammonium acetate, pH 9. Smears of fixed spermatozoa were prepared on microscopic slides, air-dried and stained by covering the slides with staining solution (0.04% Coomassie Blue – G250, 50% methanol and 10% acetic acid) for 5 minutes at room temperature, rinsed with distilled water and allowed to air-dry. Stained smears were analyzed using a Leica DMLB 100T microscope (Leica, Wetzlar, Germany), 1000x magnification. Ten to fifteen photos per slide was taken by Leica MC190 camera (Leica, Wetzlar, Germany) and LAS Ver 4.8.0 software, and up to 100 spermatozoa/slide counted to determine the acrosomal status. Blue staining in the acrosomal region of the head indicated intact acrosome, whereas spermatozoa without blue staining in acrosomal region were considered acrosome-reacted. Data were presented as the percentage of acrosome-reacted spermatozoa ± SEM.

RNA isolation and cDNA synthesis

Total RNA from spermatozoa was isolated using GenElute™ Mammalian Total RNA Miniprep Kit according to the protocol recommended by the manufacturer (*www.sigmaaldrich.com*), following the DNase I (RNase-free) treatment according to the manufacturer's protocol (www.neb.com). First-strand cDNA was synthesized using the High Capacity Kit for cDNA preparation according to the

manufacturer's instructions (*www.thermofisher.com*). Negative controls consisting of non-reverse transcribed samples were included in each set of reactions. Quality of RNA and DNA integrity was checked using control primers for *Gapdh*, as described previously by our group (for references please see [17, 64]).

Real-time polymerase chain reaction and relative quantification

The quantification of relative gene expression was done by real-time PCR (RQ-PCR) using SYBR®Green-based chemistry from Applied Biosystems (*www.thermofisher.com*) in the presence of an aliquot of 2.5 µl of the cDNA (10 ng of cDNA calculated from starting RNA) and specific primers at the final concentration of 500nM. The primer sequences used for real-time PCR analysis and Ct values, as well as GenBank accession codes for full gene sequences (*www.ncbi.nlm.nih.gov/sites/entrez*), are given in *Supplemental tables S1 to S5*. The transcription of *Gapdh* was measured in the same samples and used to correct the variations in cDNA content between the samples. Relative quantification of each gene was performed in duplicate, three times for each sample of three independent *ex vivo* experiments. All the reactions were carried out in the Eppendorf Master Cycler ep RealPlex 4 and post-run analyzes were performed using Mastercycler® eprealplex Software (for references please see[17, 64]). ***Supplemental table S1*. The primers sequences used for the real-time PCR analysis of molecular markers of mitochondrial biogenesis.**

Gene	Accession code	Primers	Primer length	Product length	Ct value
Ppargc1a	NM_031347	F: 5'-AGCCGTAGGCCCAGGTATGACA-3'<br>R: 5'-TGCTTGGCCCTTTCAGACTCCC-3'	22 bp<br>22 bp	107 bp	32.14
Ppargc1b	NM_176075	F: 5'-ACCTTCCGGTGTTCGGAGCATG-3'<br>R: 5'-GTGGAAGGAGGGCTCATTGCGT-3'	22 bp<br>22 bp	81 bp	31.21
Nrf1	NM_001100708	F: 5'-GACCATCCAGACGACGCAAGCA-3'<br>R: 5'-ATGGGCGGCAGCTTCACTGTT-3'	22 bp<br>21 bp	136 bp	28.11
Nrf2a	NM_001108841	F: 5'-AGCGGAACTGAACCGCTTGGT-3'<br>R: 5'-GTGACTGGCTGAGCAATCCCGT-3'	21 bp<br>22 bp	84 bp	27.87
Tfam	NM_031326	F: 5'-TATAGTCGTCGGCCCGAGGGAT-3'<br>R: 5'-AAGGCTGACAGGCGAGGGTATG-3'	22 bp<br>22 bp	125 bp	28.99
CytC	NM_012839	F:5'-GCAAGCATAAGACTGGACCAAA-3'<br>R:5'-TTGTTGGCATCTGTGTAAGAGAATC-3'	22 bp<br>25 bp	88 bp	24.13
Cox4i1	NM_017202	F: 5'-CGCTGAGATGAACAAGGGCACC-3'<br>R: 5'-TCCCAGATCAGCACAAGCGCA-3'	22 bp<br>21 bp	93 bp	23.01
Cox4i2	NM_053472	F: 5'-CACAGCCCAGGAAGTGCTGCTA-3'<br>R: 5'-TGTGCAGTAAGGCTCATCCGGC-3'	22 bp<br>22 bp	105 bp	33.02
Ppara	NM_013196	F: 5'-GTCCTGGAACTGAAGCGACGCT-3'<br>R: 5'-TTACGCCCAAATGCACCACGC-3'	22 bp<br>21 bp	110 bp	29.11
Ppard	NM_013141	F: 5'-ACGGTAAAGGCGGTCCATCTGC-3'<br>R: 5'-TCCTCCTGTGGCTGTTCCATGAC-3'	22 bp<br>23 bp	109 bp	26.44
Ucp1	NM_012682	F: 5'-TCAGCTCTTGTCGCCGGGTTT-3'<br>R: 5'-TGCACAGCTGGGTACACTTGGG-3'	21 bp<br>22 bp	114 bp	32.39
Ucp2	NM_019354	F: 5'-ACGACCTCCCTTGCCACTTCAC-3'<br>R: 5'-GGTACTGGCCCAAGGCAGAGTT-3'	22 bp<br>22 bp	117 bp	24.62
Ucp3	NM_013167	F: 5'-TGCTCAACCCACGGATGTGGT-3'<br>R: 5'-CCTGGCGATGGTTCTGTAGGCA-3'	21 bp<br>22 bp	112 bp	33.02
mtNd1		F: 5' GCGTGGGAGGAGCATCAGGG 3'<br>R: 5' GCGAATGGTCCTGCGGCGT A 3'	20 bp<br>20 bp	271bp	18.63
Gapdh	NM_017008	F: 5'-TGCCAAGTATGATGACATCAAGAAG-3'<br>R: 5'-AGCCCAGGATGCCCTTTAGT-3'	25 bp<br>20 bp	110 bp	22.31

Primers were designed by using software Primer Express 3.0 (Applied Biosystems) and full genes sequences from the NCBI Entrez Nucleotide database (*www.ncbi.nlm.nih.gov/sites/entrez*). F - forward; R - reverse.

Supplemental table S2. **The primers sequences used for the real-time PCR analysis of molecular markers of mitochondrial fusion and architecture.**

**Gene**	**Accession code**	**Primers**	**Primer length**	**Product length**	**Ct value**
Mfn1	NM_138976.1	F: 5'-CCTTGTACATCGATTCCTGGGTTC-3'<br>R: 5'-CCTGGGCTGCATTATCTGGTG-3'	24 bp<br>21 bp	143 bp	28.74
Mfn2	NM_130894.4	F: 5'-TCAAGCGCCAGTTTGTGGAG-3'<br>R: 5'-CACAGATGAGCAAATGTCCCAGA-3'	20 bp<br>23 bp	118 bp	27.13
Opa1	NM_133585.3	F: 5'-AAAAGCCCTTCCCAGTTCAGA-3'<br>R: 5'-TACCCGCAGTGAAGAAATCCTT-3'	21 bp<br>22 bp	101 bp	27.67
Gapdh	NM_017008	F: 5'-TGCCAAGTATGATGACATCAAGAAG-3'<br>R: 5'-AGCCCAGGATGCCCTTTAGT-3'	25 bp<br>20 bp	110 bp	22.36

Primers were designed by using software Primer Express 3.0 (Applied Biosystems) and full genes sequences from NCBI Entrez Nucleotide database (*www.ncbi.nlm.nih.gov/sites/entrez*). F - forward; R - reverse.

Supplemental table S3. **The primers sequences used for the real-time PCR analysis of molecular markers of mitochondrial fision.**

**Gene**	**Accession code**	**Primers**	**Primer length**	**Product length**	**Ct value**
Fis1	NM_001105919.1	F: 5’-ACGCCTGCCGTTACTTCTTC-3’<br>R: 5’-GCAACCCTGCAATCCTTCAC-3’	20 bp<br>20 bp	108 bp	29.66
Drp1	NM_053655.3	F: 5’-AGGTTGCCCGTGACAAATGA-3’<br>R: 5’-CACAGGCATCAGCAAAGTCG-3’	20 bp<br>20 bp	94 bp	28.92
Gapdh	NM_017008	F: 5'-TGCCAAGTATGATGACATCAAGAAG-3'<br>R: 5'-AGCCCAGGATGCCCTTTAGT-3'	25 bp<br>20 bp	110 bp	22.89

Primers were designed by using software Primer Express 3.0 (Applied Biosystems) and full genes sequences from NCBI Entrez Nucleotide database (*www.ncbi.nlm.nih.gov/sites/entrez*). F - forward; R - reverse.

Supplemental table S4. **The primers sequences used for the real-time PCR analysis of molecular markers of mitochondrial autophagy.**

**Gene**	**Accession code**	**Primers**	**Primer length**	**Product length**	**Ct value**
Pink1	NM_001106694.1	F: 5’-CAAGCAAGTGTCTGACCCAC-3’<br>R: 5’-GCTTCATACACAGCGGCATT-3’	20 bp<br>20 bp	111 bp	27.25
Prkn	NM_020093.1	F: 5’-CTTCCAGCTCAAGGAAGTGG-3’<br>R: 5’-CAGAGGCATTTGTTTCGTGA-3’	20 bp<br>20 bp	182 bp	30.25
Tfeb	NM_001025707.1	F: 5’-CGACAACATTATGCGCCTGG-3’<br>R: 5’-CTGTACACGTTCAGGTGGCT-3’	20 bp<br>20 bp	102 bp	29.83
Gapdh	NM_017008	F: 5'-TGCCAAGTATGATGACATCAAGAAG-3'<br>R: 5'-AGCCCAGGATGCCCTTTAGT-3'	25 bp<br>20 bp	110 bp	22.89

Primers were designed by using software Primer Express 3.0 (Applied Biosystems) and full genes sequences from NCBI Entrez Nucleotide database (*www.ncbi.nlm.nih.gov/sites/entrez*). F - forward; R - reverse.

***Supplemental table S5*. The primers sequences used for the real-time PCR analysis of adrenergic receptors and adrenergic receptors kinases.**

Gene	Accession code	Primers	Primer length	Product length	Ct value
Adra1d	NM_024483	F: 5'-GAAGGTGATGGGTTATGGTG-3'<br>R: 5'-GAAGCCATAGCTGAAGCCT-3'	20 bp<br>19 bp	152 bp	34.34
Adrb1	NM_012701	F: 5'-TGCCCATCCTCATGCACTGGTG-3'<br>R: 5'-GTAGGCCCGGTTGGTGACGAAA-3'	22 bp<br>22 bp	98 bp	32.96
Adrb2	NM_012492	F: 5'-CGCCCTTCAAGTACCAGAGCCT-3'<br>R: 5'-TTGCTTGTGGGTGGCACGGT-3'	22 bp<br>20 bp	131 bp	26.57
Adrbk1 (Grk2)	NM_012776	F: 5'-GCAGCGAGTGCCCAAGATGAAG-3'<br>R: 5'-CACTGCCACGCTGAATCAGTGG-3'	22 bp<br>22 bp	83 bp	27.82
Adrbk2 (Grk3)	NM_012897	F: 5'-GAGCTGACGTGCACCTTCAACG-3'<br>R: 5'-GATGGCGGCACGTGGTTTGT-3'	22 bp<br>20 bp	81 bp	28.52
Gapdh	NM_017008	F: 5'-TGCCAAGTATGATGACATCAAGAAG-3'<br>R: 5'-AGCCCAGGATGCCCTTTAGT-3'	25 bp<br>20 bp	110 bp	22.17

Primers were designed by using software Primer Express 3.0 (Applied Biosystems) and full genes sequences from NCBI Entrez Nucleotide database (*www.ncbi.nlm.nih.gov/sites/entrez*). F - forward; R - reverse.

Statistical analysis

The results of *in vivo* experiments represent group means ± SEM values of the individual variation from three independent experiments (4 to 6 rats per group). For *ex vivo* experiments data represent mean ± SEM from four to five independent replicates, from three independent experiments. Results from each experiment were analyzed by Mann-Whitney's unpaired nonparametric two-tailed test (for two-point data experiments), or by one-way ANOVA for group comparison, followed by Student-Newman-Keuls multiple range test. All the statistical analysis were done using GraphPad Prism 5 Software (GraphPad Software 287 Inc., La Jolla, CA, USA). In all cases, p-value <0.05 was considered to be statistically significant.

Reference related to Supplementary Materials and Methods

Matamoros-Volante, A. *et al*. Semi-automatized segmentation method using image-based flow cytometry to study sperm physiology: the case of capacitation-induced tyrosine phosphorylation. *Mol Hum Reprod*. **24**(2), 64-73 (2018).
